# Supplementary material for: MicroRNA Let-7a, -7e and -133a Attenuate Hypoxia-Induced Atrial Fibrosis via Targeting Collagen Expression and the JNK Pathway in HL1 Cardiomyocytes
Source: Int J Mol Sci. 2022 Aug 25;23(17):9636. doi: 10.3390/ijms23179636 (PMC9455749; doi:10.3390/ijms23179636)
Supplement: Supplementary file 1 [file ijms-23-09636-s001.zip › ijms-1824093-supplementary.pdf]

Table S1. List of differentially expressed miRNAs in hypoxic HL-1 myocyte detected by miRNA microarray assay

miRNA	Hyp/CTL (log2)	miRNA	Hyp/CTL (log2)
mmu-miR-5099	-0.77	mmu-miR-2137	0.30
mmu-let-7e-5p	-0.69	mmu-miR-1983	0.30
mmu-miR-669d-5p	-0.68	mmu-miR-711	0.31
mmu-miR-5117-3p	-0.65	mmu-miR-3474	0.31
mmu-miR-5129-5p	-0.62	mmu-miR-694	0.32
mmu-miR-1949	-0.59	mmu-miR-210-3p	0.33
mmu-miR-669a-3p	-0.58	mmu-miR-1931	0.33
mmu-miR-466f	-0.58	mmu-miR-494-3p	0.33
mmu-miR-193b-5p	-0.56	mmu-miR-700-3p	0.34
mmu-let-7a-5p	-0.53	mmu-miR-346-3p	0.35
mmu-miR-3082-5p	-0.53	mmu-miR-375-5p	0.38
mmu-miR-669c-3p	-0.52	mmu-miR-1894-3p	0.47
mmu-miR-125b-1-3p	-0.50	mmu-miR-1199-3p	0.52
		mmu-miR-5112	0.60
		mmu-miR-883a-3p	0.62

Table S2. Top 10 important pathways of differentially expressed miRNAs

pathway	p-value	miRNA numbers
TGF-beta signaling pathway	7.77E-05	12
FoxO signaling pathway	7.77E-05	14
Hippo signaling pathway	0.000214221	14
ECM-receptor interaction	0.001298154	9
cGMP-PKG signaling pathway	0.002014296	16
Circadian rhythm	0.002929838	10
mTOR signaling pathway	0.002929838	12
AMPK signaling pathway	0.003042452	14
Oxytocin signaling pathway	0.003042452	16
MAPK signaling pathway	0.013806268	18
